# Unconsciously triggered cognitive conflict influences perceptual choice in active and sedentary individuals

**DOI:** 10.3389/fnhum.2024.1400930

**Published:** 2024-06-07

**Authors:** Ruichen Jiang

**Affiliations:** ^1^School of Teacher Education, Anqing Normal University, Anqing, China; ^2^Institute of Educational Neuroscience, Anqing Normal University, Anqing, China; ^3^Affective Computing and Intelligent Learning Cognitive Psychology Experimental Center, Anqing Normal University, Anqing, China

**Keywords:** adolescents, unconsciousness, subliminal perception, cognitive conflict, perceptual choice

## Abstract

**Introduction:**

People who regularly exercise and receive training perform better when actioning unconscious cognitive tasks. The information flow triggered by a single unconscious visual stimulus has been extensively investigated, but it remains unclear whether multiple unconscious visual stimuli interact. This study aimed to explore the relationship between three simultaneous subliminal arrow stimuli (pointing in same or different directions), focusing on how they interact with each other and the subsequent priming effect on the target arrow in active and sedentary groups.

**Methods:**

We used a priming paradigm combining flanker task to test the hypothesis. A total of 42 participants were recruited. Of these, 22 constituted the active group and 20 constituted the sedentary group.

**Results:**

Behavioral data results revealed that the main effects of group and prime-target compatibility were significant. In the neurophysiological data, prime-target compatibility significantly influenced the latency of PP1. The amplitude of TP1 and TN2 mainly influenced the prime-flanker congruency. The prime-flanker congruency and groups interacted when the prime-target showed sufficient compatibility. The prime-flanker congruency, and the prime-target compatibility considerably influenced the TP3 amplitude in the anterior central frontal region (CZ electrode point).

**Conclusion:**

Event-related potentials revealed the interactions between conscious processing and subliminal conflict in the early stages of perceptual and attention processing (target-related P1 potential component). These results suggest that exercise is helpful for coping with unconscious cognitive conflict.

## 1 Introduction

The world we perceive is made up of objects and their interrelationships. More and more researchers pay attention to the relationship between physical activities and cognitive function (Güldenpenning et al., 2013; Ballester et al., 2019; Gu et al., 2019). Long-term participation in physical activities improves cognitive functions, especially in adolescents (Chaddock et al., 2011). The cognitive function of people who engage in sports is advantageous in the consciousness as well as unconsciousness fields (Jiang et al., 2021, 2023), and the advantages in processing unconscious information is not only reflected in the reaction time, but also in the neural mechanism (Güldenpenning et al., 2013; Calmels et al., 2018; Jiang et al., 2023). Due to the long-term participation in sports exercise, and the need for rapid decision-making and reaction in the sports scene, the nervous system of people who participate in regular sports may have specific changes. Therefore, people who regularly engage in sports are suitable subjects for studies on unconscious processing. Subliminal processing is a special form of unconscious processing, which refers to how the stimuli that are below the sensory threshold are processed by individuals. Although subliminal stimuli cannot be perceived by individuals, these stimuli have unconscious effects on individual behavior. Visual masking is a crucial paradigm for investigating subliminal perception (Kouider and Dehaene, 2007); the subjective visibility of a stimulus is reduced or eliminated when a masked stimulus is presented (Fehrer and Biederman, 1962; Jáskowski et al., 2003). For example, an arrow or word is visible for 33 ms when presented alone, but it is completely invisible when geometric shapes are used to mask the stimulus afterward. That is, when the duration of a stimulus is sufficiently short (≤50 ms) (Kouider and Dehaene, 2007), and it is surrounded in time and space by another stimulus, the individual awareness of this stimulus is blocked.

In terms of subliminal perception, two aspects need further investigation, namely neural basis and functional capacity. Neural basis refers to the brain mechanism of cognitive activity and functional capacity representing the depth of cognitive processing. In this study, we specifically investigated the functional capacity of subliminal processing, focusing on whether subliminal visual perception can be conflict-controlled similar with conscious visual perception, that is, whether the conflict-control system presented in conscious visual perception can also be detected in subliminal visual perception. Under conscious conditions, subjects can use top-down, task-dependent attentional control to search for specific targets in the scene while ignoring significant, bottom-up disturbing stimuli. If an unconscious conflict-control system exists, the neural potentials of unconscious conflict induced by subliminal stimuli are similar with the typical neural responses in conscious processing, as described in the literature. It may be attributed to neural activity in the anterior cingulate gyrus (Ruchsow et al., 2002).

Limited studies have explored subliminal conflict control, while some have explored the interaction or integration process of subliminal information from the holistic perspective of perception (Van Opstal et al., 2010; Faivre et al., 2014). Unconscious integration is defined as the process of generating a new representation from the unconscious processing of two or more stimuli (Mudrik et al., 2014). It includes determining the relationship between two visual or semantic representations. The idea of integrating subliminal stimuli has important theoretical implications for understanding the subliminal cognitive processes (Tu and Zhao, 2014). If the relationship among different stimuli in a priming stimulus combination can influence the response to the subsequent target stimulus, then information integration in the priming stimulus is considered to occur unconsciously, which affects the conscious processing of the target stimuli. This effect reflects in response or in neural processing. For example, by subliminally presenting several shape stimuli (Lin and Murray, 2014), arrow stimuli (Wang et al., 2017; Tu et al., 2020), letter stimuli (Van Opstal et al., 2010, 2011), lexical stimuli (Van Opstal et al., 2010; Scott et al., 2018), or facial stimuli (Liu et al., 2016), subliminal information integration can influence responses to the subsequently presented supraliminal targets. A study on visual crowding showed that a crowded priming phrase had a significant semantic priming effect on subsequent target words, even if the subliminal priming word was presented in the peripheral field of vision (Yeh et al., 2012). Thus, in few studies, three or more subliminal arrow stimuli were used simultaneously to explore unconscious conflict control, which is the focus of this study.

Literature suggests that the flanker task is one of the most commonly used experimental paradigms to reflect perceptual conflict (Eriksen and Eriksen, 1974). In the commonly used version of this task, participants are required to make quick keystrokes to the left or right directions in response to the central target arrow, while ignoring congruent or incongruent flanker interference arrows. In this task, the efficiency of cognitive control is measured by the congruency effect. That is, compared with the congruency test, the individual may show poorer behavior in the incongruency test. Typically, arrow stimuli are specific and easier to process unconsciously than lexical stimuli (Levy et al., 2014; Gelbard-Sagiv et al., 2016). Some researchers have combined the subliminal priming paradigm with the flanker paradigm to manipulate the directional relationship between the central and flanker arrow stimuli (Stock et al., 2016). Furthermore, the manipulation of the compatibility relationship between the directions of the subliminal prime arrow and the central target arrow has been used to achieve the unconscious prime-flanker congruency effect and the unconscious prime-target compatibility effect in previous studies. Studies have revealed that the event-related potential (ERP) P1 component associated with perceptual and attentional processing was sensitive to subliminal conflict only when the flanker arrow pointing was congruent with that of the central target arrow. The high processing requirements of the target stimulus may consume most of the attentional resources under the target-prime incongruent condition, resulting in insufficient processing of the prime stimulus, thereby eliminating the priming effect. Using ERP techniques, different cognitive neurophysiological sub-processes involved in information processing and response suppression can be analyzed sequentially. These findings can be interpreted based on contemporary literature. Based on this reasoning, the subliminal priming stimulus of the three arrows combined in the form of flankers adopts a top-down method for processing, that is, the unconsciously presented prime stimulus combination may trigger the conflict-control system and occupy cognitive resources. Cognitive resources are most occupied when the prime arrow is incongruent with the flanker arrow, resulting in insufficient processing of the superthreshold target stimulus, thus failing to exhibit priming effects (van Gaal et al., 2008).

The present study explored whether the pointing relationships between the subliminal prime arrow stimuli and flanker stimuli (same or different directions) would interact and integrate and thereafter influence subsequent responses to the target arrow in the active and sedentary groups. We adopted an approach similar with that used in previous studies, that is, using only the simultaneous presentation paradigm (Van Opstal et al., 2011; Lin and Murray, 2014; Wang et al., 2017). However, in this study, the three subliminal arrows were presented in a vertical flanker paradigm, which can be useful in understanding the related effects of subliminal cognitive conflict, and the results can expand our understanding of unconscious processing. Long-term exercise is associated with improved subliminal information processing ability; thus, we hypothesized that participants in active group with stronger subliminal perception ability may adequately suppress the influence of subliminal flanker stimuli under the guidance of preemptive cues, and the priming effect would be more obvious when the prime stimulus was congruent with the flanker stimulus.

## 2 Materials and methods

### 2.1 Participants

The participants were divided into active group and sedentary groups. The active group had 22 participants (average age, 20.17 years), who engaged in regular exercise (table tennis or badminton) and training 5–7 times a week for more than 1 h each time; 11 of the 22 participants were women. The sedentary group had 20 participants (average age, 20.56 years), of which eight were women, and they completed the Sedentary Behavior Questionnaire (SBQ) for adults before participating the study (Rosenberg et al., 2010). All participants had normal or corrected visual acuity, no color blindness, were right-handed, and had no history of neurological disease or surgery. We explained the safety of the device in the ERP experiment room to all participants who signed an informed consent form prior to the experiment; none of them had participated in similar experiments before. The participants were also paid for their work. The study was approved by the Special Committee for Scientific Research and Academic Ethics (Scientific Ethics) of Anqing Normal University.

### 2.2 Experimental instruments and stimulus materials

#### 2.2.1 Experimental instruments

Experimental stimuli were presented on a 17-inch HP CRT flat screen display (resolution 1,024 × 768) with a refresh rate of 60 Hz. The E-prime 3.0 software package installed on a computer was used to present the stimuli, and the response times of the participants were recorded. For all stimuli, a gray background was used (RGB: 128, 128, 128). The electrical activity of the participants’ brains was recorded through the Brain Production Recorder software by using 64 conductive electrode caps. The sampling rate was 500 Hz, the reference electrode was located in Fpz (Stock et al., 2016, 2017; Gohil et al., 2017), and the resistance between each electrode and the scalp was set to <5 kΩ.

#### 2.2.2 Experimental design and procedure

##### 2.2.2.1 Experimental design

The mixed design of 2 (groups) × 2 (prime-target stimulus compatibility) × 2 (prime-flanker stimulus congruency) was adopted, and the independent variables were as follows. (1) Group was an inter-subject variable, and the participants were divided based on whether they exercised regularly. (2) Prime-target compatibility was an intra-subject variable, referring to the compatibility relationship between the prime stimulus direction and the target stimulus direction; it had two levels, compatibility and incompatibility. (3) Another intra-subject variable was prime-flanker congruency, which refers to the congruency relationship between the prime stimulus and the flanker stimulus direction. It also had two levels, namely congruency and incongruency. The response time, error rate, average amplitude, and latency of the ERP component were considered as the dependent variables.

##### 2.2.2.2 Experimental task

**FIGURE 1:**
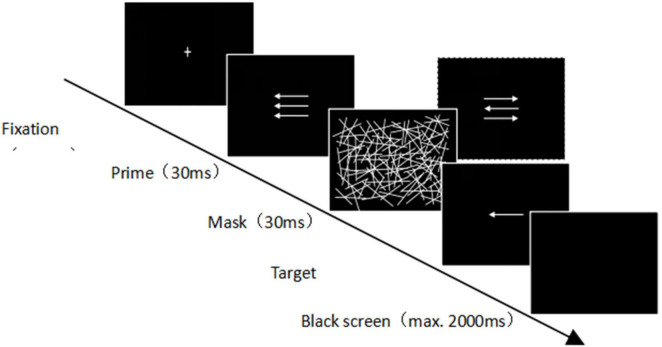
Schematics of the task combining subliminal priming and flanker interference.

Referring to the experimental paradigm of Boy et al. (2010), the experimental task was designed in the present study. The flanker task was combined with the subliminal priming task to investigate the joint effect of flanker interference on the participants’ subliminal processing. The participants were asked to press one of two keys on a custom keyboard, and all of them completed a practice session of 48 trials before the formal experiment. At the beginning of each trial, a white “cross” appeared in the center of a black background (100 ms) (Figure 1), followed by a prime in the center of the screen (a white arrow pointing left or right) and two simultaneously presented white flanker arrows both pointing left or right and located above and below the prime stimulus (30 ms). A mask (30 ms) immediately followed the prime to create a 60-ms time interval between the prime and the target; the mask comprised a randomly distributed set of white lines. After the mask stimulus, the target stimulus was presented (100 ms). The target stimulus was also a white arrow pointing left or right, and the participants were asked to press the key that corresponded to where the target arrow pointed. Each trail ended either with the first keystroke response or 2,000 ms after the target was shown (if there was no response). The reaction–stimulus interval between the participants’ previous response and the start of the next trial was controlled for a random variation of between 1,000 and 1,200 ms. The prime and target stimulus arrows pointed in the same direction in the compatible trial but in opposite directions in the incompatible trial. In addition, each trial was classified as congruent or incongruent, depending on whether the prime and flanker stimuli point was in the same or the opposite direction. There were a total of 384 trials, divided into four blocks, and different types of prime-target stimulus combinations are presented randomly. All possible combinations of prime/target stimulus compatibility, prime/flanker congruency, and target stimulus direction occurred at the same frequency. Each participant required approximately 20 min to complete this task.

### 2.3 Data analysis

#### 2.3.1 Analysis of behavioral data

(1) In the forced selection results of the discrimination task, we checked whether the results of all participants were at the random level (two-tail test, *p* > 0.05); (2) error trials, no-response trials, and those with extreme response times (i.e., <100 or >1,200 ms) were excluded. The response time and error rate were measured using 2 (group: active group; sedentary group) × 2 (compatibility: compatible; incompatible) × 2 (congruency; congruent, incongruent) repeated three-factor ANOVA. In addition, we examined whether there was a main effect or interaction among these three factors (two-tail tests, *p* > 0.05).

#### 2.3.2 Analysis of EEG data

Electroencephalography (EEG) data were processed offline using the Brain Vision Analyzer, with the following steps: (1) Filtration of the EEG data (low-pass at 30 Hz, high-pass at 0.1 Hz, slope at 24 dB/oct). (2) Removal of blink artifacts in EEG data through independent component analysis. (3) Further correction of other artifacts (e.g., maximal gradient difference between two consecutive sampling points was 50 μV; the maximum absolute value of the waveform change was 200 μV; and the minimum change value of waveform within 100 ms was 0.5 μV). (4) Segmentation of EEG data with the occurrence of target stimulus as zero point and their interception 1,000 ms backward and 500 ms forward. To avoid the influence of mask stimuli, the baseline correction was set to a time window between −500 and −200 ms (fixation point, 100 ms; prime and mask stimuli, 30 ms; and total time, 160 ms); the segmented EEG patterns were corrected on this basis. This method of baseline selection has been widely used in ERP studies under mask priming paradigm. (5) Amplitude of more than ±100 μV was considered as automatic artifact rejection. The EEG data of each participant were pre-processed according to the above steps. Finally, we averaged each segment of the prime-target compatibility and prime-flanker congruency combinations separately.

When quantifying specific ERP components, we identified the EEG components associated with the prime or target stimulus based on temporal proximity. That is, components associated with the prime appeared shortly in response to the prime stimulus, where components associated with the target appeared shortly after the beginning of the target. Subliminal priming may be biased toward early information accumulation (Scharlau and Ansorge, 2003); thus, we evaluated early attentional stimulus processing by quantifying P1 and N1 components associated with prime and target stimuli, respectively (Luck et al., 2000). P1 and N1 ERPs were quantified at Pz electrodes, where P1 and N1 induced by prime stimulus were quantified at intervals of 0–50 and 30–90 ms after target occurrence, respectively, and those induced by target stimulus were quantified at intervals of 70–110 and 90–150 ms, respectively. N2 was quantified between 180 and 300 ms, and P3 was quantified between 280 and 510 ms. Using a semi-automatic mode, the peaks of all these ERPs were automatically detected as global maximums within their respective search intervals, and if required, were manually corrected. The volatility and latency of ERP peaks were quantified at the level of a single subject and then verified using the process described in previous studies (Mückschel et al., 2014). Each electrode was compared multiple times with the average of all other electrodes using Bonferroni correction (critical threshold, *p* = 0.001). Only the electrodes with average amplitudes significantly greater than those of the remaining electrodes (i.e., negative N potential and positive P potential) were selected. The Greenhouse–Geisser method was used to correct the degrees of freedom and *p* for the statistics that did not meet the spherical tests; the LSD (Least Significant Difference) method was used for the post-tests. The above statistical analysis was performed using SPSS 26.0 statistical software. Significance was determined at *p* ≤ 0.05. Questionnaire data were analyzed using both descriptive and inferential statistical methods.

## 3 Results and analysis

### 3.1 Sample characteristics

After data collection, two and one participants from the active group and the sedentary group, were excluded, resulting in a final sample of 20 participants in the active group and 19 participants in the control group.

### 3.2 Test results for discriminating prime stimuli

After the completion of the formal experiment and the discrimination test, the participants were verbally asked whether they could see the prime stimuli; the participants reported not being able to consciously perceive the direction of the prime stimulus in most trials. The single sample *t*-tests showed that the average discernment index *d*′ in the active group was 3.2, significantly higher than the probability level (*t* = 32.00, *p* < 0.05). The average discrimination index *d*′ of the sedentary group was 2.56, which was also significantly higher than the probability level (*t* = 1.67, *p* < 0.05). The independent sample *t*-tests showed that the difference in the discrimination of prime stimuli between the active and sedendary groups was not significant (*t* = 4.03, *p* = 0.06).

### 3.3 Response time and error rate results

**FIGURE 2:**
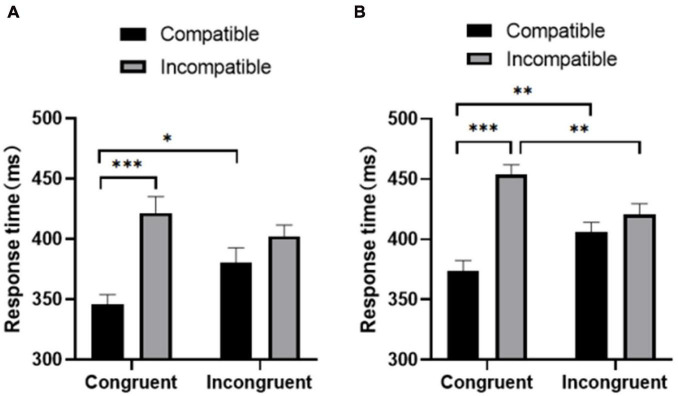
Response time of the **(A)** physically active group and **(B)** the sedentary group under congruent and incongruent conditions. *p < 0.05; ^**^p < 0.01; ^***^p < 0.001.

Behavioral and neurophysiological analyses included only the trials with the correct response between 100 and 1200 ms after the target appeared. All reported values were subjected to Greenhouse–Geisser correction, and, if necessary, Bonferroni correction. For all descriptive statistics, the standard error of mean (SEM) is presented as a measure of variability. Repeated measure ANOVA for the three factors (group, prime-target compatibility, and prime-flanker congruency) at response time (Figure 2) showed that the main effect of the group was significant [*F*(1,37) = 16.036, *p* < 0.05]; the active group responded faster to the target stimulus than the sedentary group [mean ± standard error (*M* ± SEM); physically active group: 432.955 ± 8.278 ms; sedentary group: 491.681 ± 10.535 ms]. The main effect of prime-target compatibility was significant [*F*(1,37) = 88.530, *p* < 0.001], with the participants’ response time being significantly shorter in the compatible condition than in the incompatible condition (compatibility: 443.394 ± 5.822 ms; incompatibility: 481.242 ± 6.794 ms). The main effect of prime-flanker congruency was not significant [*F*(1,37) = 0.054, *p* > 0.05].

The interaction between prime-flanker congruency and prime-target compatibility was significant [*F*(1,37) = 21.193, *p* < 0.01], and the simple main effect of prime-target compatibility was significant in both the sedentary group [*F*(1,37) = 37.851, *p* < 0.001] and the active group [*F*(1,37) = 63.086, *p* < 0.001]. However, the interactions between group and prime-flanker congruency [*F*(1,37) = 0.222, *p* > 0.05] and between group and prime-target compatibility [*F*(1,37) = 1.494, *p* > 0.05] were nonsignificant.

**FIGURE 3:**
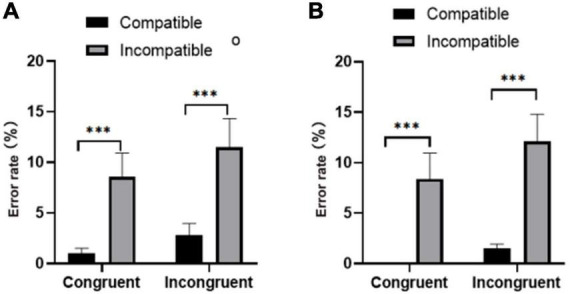
Error rates for the **(A)** physically active group and **(B)** the sedentary group under congruent and incongruent conditions. ^***^p < 0.001.

Repeated measures of the error rate (Figure 3) showed that the main effect on group factor was significant [*F*(1,37) = 6.429, *p* < 0.05]; the active group had a lower response error rate to the target stimulus than the sedentary group (*M* ± SEM); active group: 2.12% ± 0.39%; sedentary group: 5.12% ± 0.78%). The main effect of prime-flanker congruency was not significant [*F*(1,37) = 1.282, *p* > 0.05]. However, the main effect of prime-target compatibility was significant [*F*(1,37) = 13.72, *p* < 0.01], and the error rate of subjects under the compatible condition was significantly lower than that under the incompatible condition (compatibility: 2.22% ± 0.40%; incompatibility: 4.55% ± 0.61%).

### 3.4 Neurophysiological data

#### 3.4.1 Prime-induced P1 and N1 components

No significant main effect or interaction was noted in the amplitude of prime-induced P1 (PP1); however, the main effect of prime-target compatibility in the latency of PP1 [*F*(1,37) = 6.793; *p* < 0.05] was significant. The latency under compatible conditions (5.795 ± 0.458 ms) was significantly higher than that under incompatible conditions (5.529 ± 0.450 ms).

**FIGURE 4:**
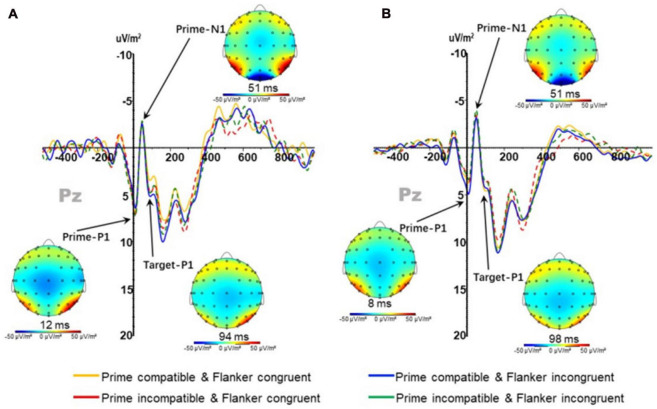
P1 and N1 components at Pz points in the **(A)** physically active group and **(B)** the sedentary group under different conditions.

There was no main effect or interaction in the amplitude and latency of prime-induced N1 (PN1); (Figure 4).

#### 3.4.2 P1 and N1 components induced by target stimulus

The amplitude of TP1 (P1 component induced by target stimuli) (Figure 4) had a significant main effect on prime-flanker congruency [*F*(1,37) = 5.812; *p* < 0.05]; the amplitude under congruent conditions (4.670 ± 0.659 μV/m^2^) was significantly lower than that under incongruent conditions (5.007 ± 0.656 μV/m^2^), and the interactions between group and prime-flanker congruencies were significant [*F*(1,37) = 7.748; *p* < 0.05]. Interactions between the prime-flanker congruency and group under prime-target compatible conditions [*F*(1,37) = 5.968; *p* < 0.05] were significant, and the amplitude under prime-flanker congruent conditions (4.278 ± 1.392 μV/m^2^) was significantly lower than that in the incongruent condition in the active group (5.852 ± 1.362 μV/m^2^), indicating that the active group participants were sensitive to subliminally presented stimuli and impacted by the flanker inference when selecting the target direction. No main effect was observed in the latency of TP1 on prime-flanker congruency. In addition, repeated measure three-factor ANOVA revealed no significant main effect or interaction in the target-induced TN1 amplitude and latency.

#### 3.4.3 Anterior central frontal lobe-N2 components

**FIGURE 5:**
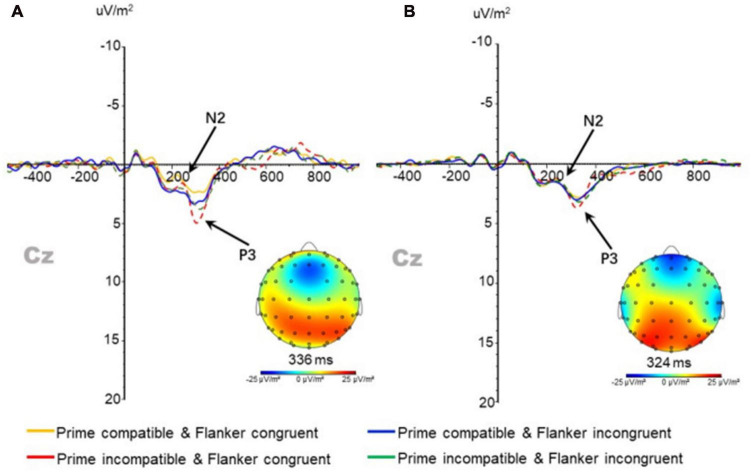
N2 and P3 components at Cz points in the **(A)** physically active group and **(B)** and sedentary group under different conditions.

In the N2 component of the anterior central frontal region (CZ electrode point) (Figure 5), the main effect of prime-flanker congruency was observed after three-factor repeated measurement ANOVA for its amplitude [*F*(1,37) = 12.364; *p* < 0.01]. The amplitude under congruent conditions (0.290 ± 0.246 μV/m^2^) was significantly lower than that under incongruent conditions (0.653 ± 0.250 μV/m^2^). The main effects and interactions of all other factors had no significant influence on the amplitude of N2 in the target stimulus. No main effect or interaction was found for the latency in the repeated measure three-factor ANOVA.

#### 3.4.4 P3 components

Repeated measure three-factor ANOVA for P3 peak amplitude and latency in the anterior central frontal region (CZ electrode point) (Figure 5) revealed a significant main effect in the prime-target compatibility of the amplitude [*F*(1,37) = 18.099; *p* < 0.01]. The amplitude under compatible conditions (3.231 ± 0.234 μV/m^2^) was significantly lower than that under incompatible conditions (3.900 ± 0.276 μV/m^2^). No significant main effect or interaction was found in the latency.

## 4 Discussion

Although research has made great progress in explaining the information flow triggered by a single unconscious visual stimulus, whether there are interactions between multiple unconscious information sources remains debatable (Van Opstal et al., 2010; Faivre et al., 2014). In this study, we used priming paradigm and EEG to explore the subliminal conflict control in the active and sedendary groups. Analysis of behavioral and ERP data of PP1 revealed a significant priming effect for both active and sedendary groups under congruent and incongruent conditions. However, TP1 was impacted when flanker stimulus and prime were incongruent. The results of this study indicated that in a laboratory setting, flanker stimuli surrounding the prime could cause subliminal cognitive conflict (Eriksen and Eriksen, 1974). This conflict was detected by changes in the neural activity of an individual in response to a target stimulus, and similar findings have been reported in previous studies on consciousness (Boy et al., 2010; Wu et al., 2015). One study used the paradigm combining the supraliminal flankers around the target stimuli and subliminal prime; target-related P1, which is related to perception and attention processing, was sensitive to subliminal priming only in the prime-flanker congruency condition (Jiang et al., 2023). Under the condition of prime-flanker incongruency, the higher processing requirements of the target stimulus may consume most of the attention resources, resulting in the prime stimulus being under-processed, thus decreasing or even eliminating the priming effect (Clayson and Larson, 2011; Kuratomi and Yoshizaki, 2013; Stock et al., 2016). Because of the limited processing power of our attention system, cognitive conflict or interference occurs when a particular feature of a stimulus is incompatible or incongruent with the properties of the surrounding stimulus (Zysset et al., 2001).

Furthermore, for both active and sedendary group participants, the response time of the subjects was the longest when the prime-flanker was congruent and the prime target was incompatible. Neuroelectrophysiology results showed that in the prime-flanker incongruent condition, the participants consumed the most cognitive resources, indicating that the active and sedendary group participants could analyze and process the combination of subliminal prime-flanker stimuli as a whole despite the presence of forward cues when performing subliminal perception. A reasonable explanation for these findings lies in the unconscious integration effect (Faivre et al., 2014; Lin and Murray, 2014). When the prime-flanker was congruent and the prime target was incompatible, the participants perceived the greatest conflict when responding to the target stimulus because they took the prime and flanker stimuli as a whole, which is referred to as the integration effect. Subsequently, the perception of conflict was strongest when the target stimulus was processed because the target stimulus differed from the previous three stimuli. Most researchers have adopted the subliminal priming paradigm. For example, relationships between simultaneously presented subliminal prime stimuli have been found to influence subsequent responses to target stimuli presented above the threshold (Van Opstal et al., 2011; Faivre et al., 2014; Lin and Murray, 2014; Van Gaal et al., 2014; Celeghin et al., 2015; Liu et al., 2016; Wang et al., 2017). Because an arrow is a simple semantic symbol, its pointing relationship is concrete, and its interpretation in the unconscious processing is easier than that of words (Gelbard-Sagiv et al., 2016). Perceptual selection and grouping of unconscious stimuli depend on several situational factors, such as perceptual load (Lavie, 2005) or spatial proximity (Diede and Bugg, 2016). In the early stages of visual processing, low-level perceptual grouping based on factors, such as direction, color (Bortolotti et al., 2022), and motion (Mozer and Vecera, 2005), can modulate attention to stimuli throughout the visual field. In addition, the amplitude of prime-flanker congruent condition was significantly lower than that in the incongruent conditions in the active group, indicating that the participants in this group were sensitive to subliminally presented stimuli and impacted by the flanker inference when selecting the target direction.

Another important theoretical issue highlighted in this study is the relationship between cognitive control and consciousness (Desender and Van den Bussche, 2012; Ansorge et al., 2014). According to classical theories, such as the global neural workspace theory, cognitive control is exclusive to the consciousness domain (Dehaene and Naccache, 2001; Martens et al., 2011). A typical manifestation of cognitive control is the adaptation to response conflict, where an individual adapts to conflicts by reducing the impact of irrelevant information; an interesting question here is whether this is also possible when the conflict itself is unconscious. In our study, attention to specific stimulus features associated with target judgment (the direction of the target arrow) probably led to an increase in cortical activation in the visual region to similar stimuli that are unconsciously perceived, suggesting that both subliminal primes and flankers can induce unconscious executive control effects (Almeida et al., 2008). In the sequential presentation paradigm, the effectiveness of subliminal prime stimuli presented separately reflects bottom-up response activation, rather than top-down executive control. Although the priming effect is obvious, when multiple subliminal primes are presented simultaneously, conflicts suppressed by visual masking can affect the responses to targets among active individuals (Peremen and Lamy, 2014). In addition, the interpretation of executive control in information theory attributes the conflict effect to the increased uncertainty of information related to a specific target (Fan et al., 2014). For example, in the flanker task, the prime and flanker arrows pointing in the same direction can be grouped together, increasing the level of uncertainty associated with the central target stimulus, eventually triggering the executive control of attention. Thus, the findings of this study seem to challenge earlier theories that emphasize the limitations of unconscious information in realizing advanced information processing (Dehaene and Naccache, 2001; Kiefer, 2012).

Interestingly, this study also found that the location of the prime and target stimuli is cued, and the combination of prime and flanker stimuli can affect the behavioral performance of subjects. An inverse response time pattern may emerge. We believe that top-down attention, rather than bottom-up attention, facilitated the processing of unconscious stimulus combinations in the current experiment (Naccache et al., 2002; Sumner et al., 2006). Studies have indicated that the top-down attention process may improve the ability to retain the fleeting information carried by unconscious stimuli. Our results, therefore, add to the growing body of literature, suggesting that many aspects of cognitive control may not be uniquely linked to consciousness (Kunde et al., 2003; Pavone et al., 2009).

## 5 Limitations of the study

A systematic study of the limited spatio-temporal scope of unconscious information integration is required to comprehensively understand the unconscious executive control processes. In some studies, the conflict effect has been shown to be more obvious when the distractor is presented briefly before the target object. Future research can further explore the processes that trigger the unconscious visual interaction or integration of stimulus combinations at different time intervals. In addition, the discovery of unconscious information integration can help better understand the global neural workspace theory and various theories of consciousness. Finally, we suggest that future studies introduce target and flanker stimuli with emotional associations (i.e., excitement or threat) to assess whether unconscious executive control depends primarily on the nature of the stimulus.

## 6 Conclusion

Although some alternative explanations for our current results are worth exploring in the future, as discussed above, it is preferable to interpret the data in terms of conflict control. When conflict information was unconscious, participants from both active and sedendary groups exhibited good cognitive control; however, at the neuroelectrophysiological level, the active group participants appeared to be affected by the conflict, suggesting that they are more sensitive to subliminal unconscious stimuli.

## Data availability statement

The original contributions presented in this study are included in this article/supplementary material, further inquiries can be directed to the corresponding author.

## Ethics statement

The studies involving humans were approved by the Special Committee for Scientific Research and Academic Ethics (Scientific Ethics) of Anqing Normal University. The studies were conducted in accordance with the local legislation and institutional requirements. The participants provided their written informed consent to participate in this study.

## Author contributions

RJ: Conceptualization, Data curation, Formal analysis, Funding acquisition, Investigation, Methodology, Project administration, Resources, Software, Supervision, Validation, Visualization, Writing – original draft, Writing – review & editing.
